# Validation of Normal P-Wave Parameters in a Large Unselected Pediatric Population of North-Western Romania: Results of the CARDIOPED Project

**DOI:** 10.1155/2021/6657982

**Published:** 2021-03-06

**Authors:** Gabriel Cismaru, Cecilia Lazea, Lucian Mureşan, Gabriel Gusetu, Radu Rosu, Dana Pop, Dumitru Zdrenghea, Anca Daniela Farcaş, Simona Sorana Căinap

**Affiliations:** ^1^5th Department of Internal Medicine, Cardiology Rehabilitation, “Iuliu Hatieganu” University of Medicine and Pharmacy, Cluj-Napoca, Romania; ^2^Department of Pediatrics I, Emergency Clinic Hospital for Children, “Iuliu Hatieganu” University of Medicine and Pharmacy, Cluj-Napoca, Romania; ^3^Cardiology Department, “Emile Muller” Hospital, 68100 Mulhouse, France; ^4^Emergency Clinical County Hospital, 40006 Cluj-Napoca, Romania; ^5^Department of Pediatrics II, Emergency Clinic Hospital for Children, “Iuliu Hatieganu” University of Medicine and Pharmacy, Cluj-Napoca, Romania

## Abstract

**Aims:**

Reference values of the P-wave on 12 lead electrocardiograms are lacking for children and adolescents in Eastern Europe. Hence, the present study is aimed at determining the standard values of the P-wave in children and adolescents based on ECG data from the CARDIOPED project, a large-scale general population of children who participated in a screening program in Transylvania, Romania.

**Methods and Results:**

A total of 22,411 ECGs of participants aged 6 to 18 years old from a school-based ECG screening were obtained between February 2015 and December 2015 in Transylvania, Romania. Three pediatric cardiologists manually reviewed each ECG. P-wave duration, voltage, axis, and correlation with gender and age were analyzed. The mean P-wave duration was 88 ± 10.7 ms, with a maximum duration of 128 ms. P-wave showed a positive correlation with age but did not differ between sexes. There was a positive correlation between the P-wave duration and the heart rate, but not with the body max index. The mean P-wave axis was 40.4 ± 31.1, and the mean P-wave amplitude was 0.12 ± 0.03 mV.

**Conclusion:**

In this study on many pediatric subjects, we have provided normal limits for the P-wave in Romanian children aged 6-18 years. Our findings are useful for creating interpretation guidelines for pediatric ECG.

## 1. Introduction

A correct interpretation of electrocardiograms in children relies on comparison with standard values derived from the normal population. Comprehensive data on ECG in Romania are lacking. No study to date explores P-wave characteristics in Eastern Europe and, more specifically, in Romania. Romania is a south-eastern country of Europe and a state member of the European Union. It shares borders with Hungary, Serbia, Bulgaria, Ukraine, and the Republic of Moldova. It covers 238,397 km^2^ and has 19.71 million inhabitants with a median age of 40.9 years. ECG interpretation depends on knowledge of normal limits, which in children are age-dependent. Diagnostic ECG criteria require the availability of appropriate normal references.

A P-wave duration of more than 110 ms was associated in adults with left atrial enlargement, electromechanical dysfunction, atrial fibrillation, and embolic stroke [1–6]. In pediatrics, the currently accepted “normal” P-wave is 70 ms for infants and 90 ms for children [7, 8].

Our study is aimed at determining the P-wave's standard values in a large nonselected population of healthy children from North-Western Romania.

## 2. Material and Methods

The study population consisted of 23,833 healthy children consecutively recruited from the primary schools of North-West of Romania. We eliminated 1422 ECGs because of ECG limb lead reversal, inappropriate attachment of electrical leads, artifacts/drifts, ectopic atrial rhythms, or junctional rhythm. In consequence, 22,411 ECS were considered for the final analysis. Twelve primary schools randomly selected from 64 schools in Transylvania were approached for participating in this study. Ethical permission was granted by the Ethics Committee of the “Iuliu Hatieganu” University of Medicine and Pharmacy and agreement from the schools, head teachers, school nurses, generalists, and parents of the children. Written consent from the parents was obtained. They were told that the project was aimed at estimating the frequency of cardiac disease in school children. They were asked to complete some demographic data like weight, height, urban or rural area of living, and parents' cardiac disease. No children had a history of cardiovascular disease, and none received medication. All 22,411 children had a normal physical examination.

All children underwent a 12-lead digital ECG recording using 20 machines BTL-08 MT Plus at a sampling rate of 2000 Hz. The frequency response of this recorder is flat to 170 Hz. ECGs were analyzed by 200 physicians (cardiologists or pediatricians) for three months and manually reviewed by 3 cardiac pediatricians for three years. The onset and the offset of the P-wave were defined as the junction between the P-wave and the isoelectric line before and after the wave's start. Amplitude measurements of the P-wave were made using the PR segment as the baseline. The 200 physicians could compare the values manually obtained from magnified ECG tracing on the monitor with the computer program's values on the digitized ECG. When there was a difference between manual and computer measurement, the manual value was selected for further statistical analysis.

### 2.1. Statistical Analysis

Continuous variables are expressed as mean ± standard deviation and were compared with the two-sided Student test (*t*-test). Categorical variables are expressed as counts or percentages and were compared using the chi-square test. Linear regression analysis was used to test the prediction of P-wave duration by age and heart rate. Multiple imputations were used for the missing data. A *p* value of < 0.05 was considered statistical significance. All statistical analyses were performed using the SPSS 23.0 software (SPSS In. Chicago, IL, USA).

#### 2.1.1. Dealing with Missing Data

Among the 22,411 children used in cross-sectional analyses, there were small amounts of missing data for height, weight, and P-wave characteristics. These data varied from 0 (e.g., for child age and gender) to 4% (for the P-wave duration, amplitude, and axis) and 8% (for weight, height, and BMI where the parents offered data). We used multiple imputations for the missing data to increase power and minimize selection bias in our findings.

## 3. Results Population

Demographic characteristics are presented in Table 1. In brief, they were 22,411 children with ages ranging from 6 to 18 years old (corresponding to the eight grades of primary school), weight from 16 to 135 kg, height from 39 to 207 cm, and BMI from 14 to 50 kg/m^2^.

Of the 22,411 children, 22,349 had normal sinus rhythm. Heart rate ranged from 45 to 168, with a mean of 88.2 ± 11.5 bpm. The mean QRS axis was 62.1 ± 29.3° with the axis being 0 to 120° in 96.4% of the children. The mean PR interval measured 144.8 ± 24.5 ms. The mean QRS duration was 81.3 ± 10.8 ms with 99.9% of the children having a QRS duration of <120 ms. The mean QT duration was 408 ± 27.9 ms.

### 3.1. P-Wave Description

**Figure 1:**
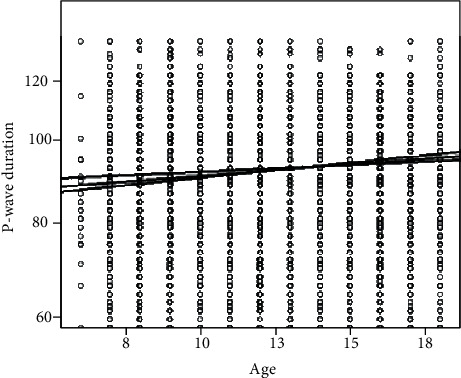
The positive correlation between the P-wave duration and the age (scatterplot with regression line; standardized beta coefficient = 0.095; *p* < 0.000001).

**Figure 2:**
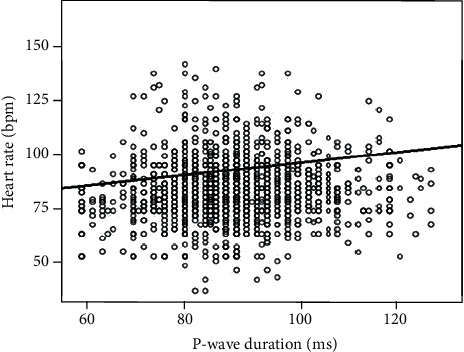
The positive correlation between the P-wave duration and the heart rate (scatterplot with regression line; standardized beta coefficient = 0.08; *p* < 0.000001).

The mean values of P-wave duration were 88.2 ms with a minimum of 50 ms and a maximum of 128 ms (Table 1). P-wave duration was correlated to age (*r* = 0.075, *p* < 0.001), indicating a progressive increase of P-wave duration with increasing age (Figure 1). There was no significant difference between sexes (88.0 ± 10.6 ms in boys vs. 87.8 ± 10.6 ms in girls, *p* = 0.051). Furthermore, P-wave duration showed a statistically significant correlation with heart rate (*r*^2^ = −0.095, *p* < 0.001) (Figure 2), age (*r*^2^ = 0.075 with *p* < 0.001), weight (*r*^2^ = 0.044 and *p* < 0.015), and height (*r*^2^ = 0.063 and *p* < 0.001) but not with the BMI (*r*^2^ = 0.022; *p* = 0.216).

**Figure 3:**
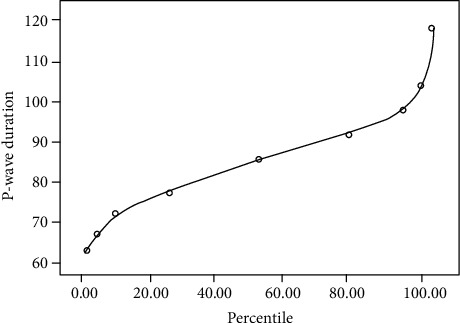
Second, 10th, 25th, 50th, 75th, 95th, and 98th percentiles for the P-wave duration.

The 95th and 99th percentiles for the P-wave duration were 106 and 120, respectively (Table 2). The 95th and 99th taken as the upper limit of normal are arbitrary cut-off values frequently used in the pediatric population for hypertension, obesity, electrocardiogram, or echocardiogram parameters. The 2nd percentiles, taken as the lower limits of normal, were 66 ms (Figure 3).

Using the 90 ms cut-off value for increase duration of the P-wave, 31% of our population would have been classified as having an increased value.

The amplitude of the P-wave was 0.12 ± 0.03 mV with a range between 0 and 0.25 mV. We did not find a significant association between P-wave amplitude and heart rate, sex, age, weight, height, and BMI (all *p* values > 0.05).

P-wave axis was measured using the positive or negative deflections in all 6 limb leads and calculating the direction of electric activity on the hexaxial reference system. P-wave axis had a mean of 40.4 ± 31.1°. There was no correlation between the P-wave axis and other variables like heart rate, age, sex, weight, height, and BMI.

## 4. Discussion

Standards of normal values for ECG interpretation in normal children have been available since 1979 [9]. Davignon et al. recorded 2141 ECGs in children from Quebec, Canada, and developed graphs and tables of normal values for future use when evaluating ECG in pediatric population. Recent studies suggest that some of these cut-offs should be reviewed and maybe revised to consider the newer research on larger populations of children, as possible physiological changes in children and races that might have appeared since the original paper was published.

Macfarlane et al. [10] showed that the 98th percentile of the normal amplitude in children could be out of range in 46% of patients when compared with values obtained by Davignon et al. Furthermore, Rinjbeeck et al. [11] showed on European population the differences in normal values when compared with those obtained by Davignon and Macfarlane. Older normal limits may no longer apply to current pediatric practice [12].

In 1990, the American Heart Association recommended a minimum of 500 Hz which has been recommended for sampling rate in adult ECG [13]. As for pediatric ECGs, higher sampling rates should be used [8, 14]. In the study of Davignon et al., ECGs were recorded at a sampling rate of 333 Hz. Later, Macfarlane et al. used a sampling rate of 500 ms and found that 46% of the amplitude measurements were beyond the cut-off values recommended by Davignon. Our study applied a sampling rate of 2000 Hz, which was considered sufficiently high to record a pediatric ECG accurately.

In a study on 232 healthy children, Kose et al. [15] demonstrated that the increase in P-wave duration corresponded to age increase in a cohort aged 7 to 15 years. In a later study [16], P-wave duration was also associated with age in hospitalized children, with the most significant increase occurring at >10 years of age. In the study of Loo et al. [16], the prevalence of large P-waves compared to the cut-off of 90 ms is particularly high (27%) and in opposition with the low percentage of atrial arrhythmias in this pediatric group.

Investigations on African population [17] found a P-wave duration of 70 ms in a cohort of 1500 children aged 0 to 12 years. Probably, the difference compared to our values comes from the fact that we also included children \between 12 and 18 years. It is well known that the duration of the P-wave increases with age, which is why in our study, the average duration of the P-wave was 88.2 ms which is higher compared to the value found on the Nigerian population.

A study performed in Turkey on children up to 16 years of age found an average P-wave duration of 64 ms in girls and 62 ms in boys. Besides the fact that this study included a 10-times smaller number of children compared to our study, it also included newborns, infants, and children aged 1 to 6 years. We believe that the difference with our results is due to a shorter duration of the P-wave in newborns and infants, as the P-wave duration is shorter in smaller ages [18].

Another European study [11], similar to ours, was performed on Dutch population and obtained values for the P-wave duration higher than ours: 92 ms for the age group 5-8 years, 98 ms for 8-12 years, and 100 ms for 12-16 years. In Rinjbeck's study, the weight and height of the children are not specified. It is possible that the differences observed between the 2 studies in the duration of the P-wave are related to the difference in weight and height between the Dutch [19] and the Romanian population [20].

Research performed on American population [21] found P-wave duration values similar to our values in Caucasian individuals. On the other hand, African-American individuals had a longer P-wave duration compared to ours, and also higher than the values found in African individuals in the study of Kolawole et al.

ECG recordings on a Japanese population of children found P-wave duration values similar to those recorded in our group of Caucasian children: 77 ms for 1st graders, 87 ms for 7th graders, and 99 ms for 10th graders. There were no significant differences in age or sex distribution between our study and that of Yoshinaga et al. [22]. The number of children was high in both studies.

Prolonged P-wave duration has been described with different pediatric medical conditions. One of the most important pediatric pathology remains cancer, where excellent long-term survival could raise more problems such as chemotherapy induced cardiomyopathy [23]. Ozmen et al. [24] compared 43 pediatric patients with pulmonary stenosis to 33 healthy pediatric controls and showed increased P-wave duration in the first group. Furthermore, Ho et al. [25] compared 94 children with ostium secundum atrial septal defect with healthy children. They observed an increase in the mean P-wave duration in patients with the atrial septal defect. Wong et al. [26] also demonstrated an increase in the P-wave duration in patients with Fontan surgery compared to healthy children matched for age and sex. Also, the P-wave's increased duration was noted in patients with tetralogy of Fallot [27] and viral infections [28, 29]. Probably, the 90 ms value is correct for children who have congenital heart disease or atrial arrhythmias. But for healthy children, the cut-off value should be revised.

The interatrial block in adults is defined as a prolongation of the P-wave >110 ms on standard 12-lead ECG. In children, cut-offs for P-wave durations are lower, 90 ms, due to reduced myocardial mass in the pediatric population. However, in children, an increase in P-wave duration is, in fact, proportionate to age. In our study, we found that the duration of the P-wave in healthy children had a mean of 88.0 ms and was positively correlated to age; therefore, it increases with the age of the individual, as reported earlier [24]. Thirty-one percent of our population would have been classified as having an increased value when using the 90 ms cut-off to increase the P-wave duration. The 95th and 99th are arbitrary cut-off values frequently used in the pediatric population for electrogram characteristics and hypertension, obesity, and echocardiogram values. The 95th and 99th percentiles for the P-wave duration in our pediatric population were 106 and 120, respectively; therefore, the 90 ms cut-off value proposed for the interatrial block in the pediatric population should be reconsidered.

## 5. Limitations

Our physicians used manual P-wave measurements on a magnified screen image. Magnification of ECGs on a high-resolution screen may differ from manual measurements on paper-printed ECGs but can save time. All ECGs were analyzed for three months.

## 6. Conclusion

In this study on a large unselected pediatric population, we have provided limits for the P-wave in Romanian children aged 6-18 years. The mean P-wave duration was 88 ± 10.7 ms, with a maximum duration of 128 ms. P-wave duration showed a positive correlation with age and heart rate.

## Figures and Tables

**Table 1:** Patient characteristics.

Parameter	
*N*	22,411
Age (years; mean ± SD)	12.4 ± 3.1
Gender (male; *N* %)	10,712 (47.8%)
Age distribution	
6-7 years	48 (0.2%)
7-8 years	1237 (5.6%)
8-9 years	1771 (8.0%)
9-10 years	1806 (8.2%)
10-11 years	2075 (9.4%)
11-12 years	2314 (10.5%)
12-13 years	2080 (9.4%)
13-14 years	2079 (9.4%)
14-15 years	2082 (9.5%)
15-16 years	2224 (10.1%)
16-17 years	1940 (8.8%)
17-18 years	1580 (7.2%)
18 years	792 (3.6%)
Heart rate (bpm; mean ± SD)	88.2 ± 11.5
Mean P-wave duration (ms; mean ± SD)	88.0 ± 10.7
Mean P-wave axis (grades; mean ± SD)	40.4 ± 31.1
Mean P-wave amplitude (mV; mean ± SD)	0.12 ± 0.03
Weight (kg; mean ± SD)	49.1 ± 16.8
Height (cm; mean ± SD)	154.8 ± 16.0
BMI (kg/m^2^; mean ± SD)	20.0 ± 4.0

**Table 2:** The 95th and the 99th percentile of the P-wave duration, amplitude, and axis.

Number of patients = 22,411	95th percentile	99th percentile
P-wave duration	106	120
P-wave amplitude	0.18	0.25
P-wave axis	75	96

## Data Availability

The data used to support the findings of this study are included within the article.
